# The high price that Colombia has paid for its lack of biotechnological sovereignty

**DOI:** 10.1016/j.lana.2023.100560

**Published:** 2023-07-17

**Authors:** Camilo Guzman T, Salim Máttar, Nelson Alvis-Guzmán, Fernando De la Hoz

**Affiliations:** a University of Córdoba, Institute of Biological Research of the Tropics, Monteria, Colombia; b Alzak Foundation, Cartagena, Colombia; c University of Cartagena, Cartagena, Colombia; d Department of Public Health, National University of Colombia, Bogota, Colombia

During the COVID-19 pandemic, Latin American countries suffered the collapse of their health systems. This was caused by the high demand for care for those infected, which in parallel was added to the care of patients with other types of diseases. The excess demand for health services caused medical and laboratory supplies to decline rapidly. In this sense, the shortage was a reality, and thus, imported molecular diagnostic equipment, supplies, and kits soon became scarce. The COVID-19 pandemic disclosed a health crisis caused by an insufficient systematic policy of appropriating scientific knowledge. Without a model of scientific knowledge, there are few possibilities for consolidating markets for technologies such as diagnostic methods for new infectious agents, medicines, vaccines, and medical devices, among others.

The effects of this asymmetry in the availability and access to technologies and biotechnologies were essential for many countries, especially low-income countries, to suffer high rates of excess mortality from COVID-19. A systematic review and meta-analysis on excess mortality from COVID-19 from 79 countries found an overall excess mortality of 104.84 (95% CI: 85.56–124.13) per 100,000 inhabitants. South America had an excess mortality rate of 30% above the global average; developing countries had twice the rates than developed ones (135.80 vs. 68.08). Lower-middle-income and upper-middle-income countries (133.45 vs. 149.88, respectively) had twice the excess mortality of high-income countries.^1^^,^^2^ In Colombia, excess mortality attributable to COVID-19 was between 15% and 20%.^2^ Colombia is in third place in South America with 6,372,392 total cases. The country ranked third in the region in mortality rate due to COVID-19, with the highest number of deaths (n = 142,807); 2772 deaths/1 million inhabitants) (https://www.worldometers.info/coronavirus/#countries, accessed June 30, 2023).^3^

During the COVID-19 pandemic, it became clear that rich countries had priority access to vaccines; this "VIP" treatment was caused by the monopoly of the large pharmaceutical companies that distributed the vaccines then. The pandemic demonstrated, in the lower-middle-income countries of Latin America, little investment in research, development, and innovation activities, which shows an imbalance in the levels of competitiveness. We pay dearly in the pandemic for dependence on other countries. It was demonstrated that Latin American countries must develop systematic and continuous strategies based on research, development, and innovation activities at their different maturity levels that allow increasing the number of patents and biotechnological developments to shape a universal public health crisis better.

Scientific autonomy is justified because it promotes progress and benefits society. Access to medicines and health supplies is essential to achieve total health coverage. The increase in costs and the scarcity of medicines are some problems that third-world countries face.^4^ Significant social, economic, and environmental achievements can be obtained if Latin America decisively assumes the challenge of defining a parallel health and science, technology, and innovation policy that leads to biotechnological sovereignty. There is currently a slowdown in the Latin American economy that poses fundamental challenges in social matters. During the pandemic, monetary poverty in countries like Colombia increased from 35.7% in 2019 to 39.3% in 2021 and 44.6% in rural areas.^5^^,^^6^

In Colombia, there have been restrictions on scientific autonomy and biotechnological self-sufficiency for more than 50 years to favor commercial interests that have not benefited the nation. The country's health community remembers the wrong decision to close the National Institute of Health biological production plant at the end of the 90s. The erroneous form of the government of those times in making scientific decisions suffocated scientific autonomy, and it caused the country's deaths and cost overruns by having to import biologicals subsequently.^7^ Adopting a free trade model does not justify the loss of autonomy of society and the dependence of the exercise of Colombian scientists on imported inputs.

Biotechnological sovereignty is not a mere nationalist concept; it is an ecosystem allowing medicines at a lower cost, accompanied by the Ministries of Health and Science technology policies. With the support of the state and a solid pharmaceutical policy, the country could manufacture the most widely used medicines to treat the most prevalent diseases. Among these drugs are antihypertensives, analgesics, hypoglycemic agents, antibiotics, antiretrovirals, anticancer drugs, vaccines, diagnostic means, and insulin.

Biotech sovereignty is not a dream of third-world countries. An example of biotechnological independence has been given by Cuba, China, Russia, and India, among others, who could design and manufacture anti-COVID-19 vaccines. It has been denoted the biotechnological resounding failure of sovereignty in Colombia. Scientific autonomy based on innovation processes that strengthen biotechnological autonomy contributes to the economy through gross added value, high-quality employment, appropriation and social dissemination of knowledge, and cost reduction.

## Contributors

All authors equally participated in articulating ideas, writing, and discussing the manuscript.

## Declaration of interests

The authors have no conflict of interest.
